# The Role of Occupational and Environmental Exposures in the Pathogenesis of Idiopathic Pulmonary Fibrosis: A Narrative Literature Review

**DOI:** 10.3390/medicina54060108

**Published:** 2018-12-10

**Authors:** Samuel P. Trethewey, Gareth I. Walters

**Affiliations:** 1 Respiratory Medicine, University Hospitals Birmingham NHS Foundation Trust, Birmingham B95SS, UK; s-trethewey@doctors.org.uk; 2 Birmingham Regional NHS Occupational Lung Disease Service, Birmingham Chest Clinic, Birmingham B33HX, UK; 3 Occupational and Environmental Medicine, Institute of Clinical Sciences, University of Birmingham, Birmingham B152TT, UK

**Keywords:** idiopathic pulmonary fibrosis, IPF, pathogenesis, occupational, environmental, exposures

## Abstract

Idiopathic pulmonary fibrosis (IPF) is a chronic interstitial lung disease characterised by a progressive and irreversible decline in lung function, which is associated with poor long-term survival. The pathogenesis of IPF is incompletely understood. An accumulating body of evidence, obtained over the past three decades, suggests that occupational and environmental exposures may play a role in the development of IPF. This narrative literature review aims to summarise current understanding and the areas of ongoing research into the role of occupational and environmental exposures in the pathogenesis of IPF.

## 1. Introduction

Idiopathic pulmonary fibrosis (IPF) is a chronic interstitial lung disease characterised by a progressive and irreversible decline in lung function, which is associated with poor long-term survival [1]. Cardinal signs and symptoms of IPF include chronic dyspnea, which is worse on exertion, a persistent cough, bilateral inspiratory crackles on chest auscultation and finger clubbing [2]. The rate of clinical decline seen in patients with IPF is highly variable. Many patients with IPF will experience a slow but progressive decline in their clinical condition, whereas a small proportion will experience rapid deterioration as part of an accelerated clinical course punctuated by acute exacerbations [3]. The rate of acute exacerbations in patients with IPF has been estimated to be between 10 and 20% of patients per year [4].

The incidence and prevalence of IPF varies between studies, which likely reflects historical differences in diagnostic criteria [5]. IPF can no longer be considered a rare disease and the incidence and prevalence of IPF appears to be increasing worldwide [6]. It is unclear whether the observed increase in incidence reflects a true increase or is just due to improved identification of cases as a result of higher resolution imaging, greater awareness of the condition, a lower threshold for diagnosis, changes in diagnostic criteria or improved multi-disciplinary team (MDT) input [7].

In a large systematic review of 34 studies evaluating global IPF incidence and mortality, Hutchinson et al. [8] estimated the overall incidence of IPF as between 3 and 9 cases per 100,000 population per year in Europe and North America, with lower incidence rates noted in South America and East Asia. A recent analysis of UK population-based data, between 2000 and 2012, found that the incidence of IPF has increased over time, with an overall incidence rate of 2.85 to 8.65 per 100,000 patient-years (depending on whether a narrow case-definition or a broad case-definition of IPF was used) [9]. Similar incidence rates have been observed in the US [10] and in Italy [11], whilst a slightly higher incidence rate has been observed in the Canadian population [12].

Importantly, patients with IPF experience significant morbidity and mortality. Based on the most recent analysis of UK data, the average life-expectancy in patients with IPF is around 3 years [9]. Crucially, the authors noted that there was no increase in survival in patients with a diagnosis of IPF during the study period. Furthermore, long-term survival was remarkably similar to a previous analysis of UK data which gave a 5-year survival of approximately 40%, suggesting that long-term survival has remained relatively static over recent years [13]. In an observational study of the World Health Organisation mortality data from 2001–2013, Marshall et al. [6] identified wide variation in mortality rates across Europe with an increase in mortality over time in most European countries. There are several possible competing factors which may explain the finding that mortality in IPF is static, or in many cases, worsening over time. An increase over time in the number of patients being labelled as having IPF, for reasons previously discussed, may have the direct effect of more deaths being attributed to the disease, giving a higher rate of death per 100,000 population [7]. The impact of the two licensed antifibrotic treatments, Pirfenidone and Nintedanib, on long-term survival in IPF is yet to be determined but current evidence of a survival benefit is promising [14].

The poor prognosis seen in patients with IPF necessitates timely diagnosis to identify patients who might benefit from treatment targeted at maintaining lung function. However, diagnosing IPF remains a complex and challenging endeavour, which requires detailed history taking, specialist investigations and MDT input. Often, IPF has an insidious onset and many patients show symptoms as early as 5 years before the diagnosis is made [15].

## 2. The Diagnostic Challenge

Historically, IPF has been a challenge to diagnose due to an incomplete understanding of the disease pathogenesis, inconsistent diagnostic criteria and a lack of international agreement [16]. IPF is a diagnosis of exclusion, made by the MDT, in patients with evidence of interstitial lung disease in the absence of identifiable causes.

The most recent international consensus guideline for the diagnosis of IPF has been produced via collaboration between the American Thoracic Society, the European Respiratory Society, the Japanese Respiratory Society and the Latin American Thoracic Association (ATS/ERS/JRS/ALAT) [17]. Key radiological features required for the diagnosis of IPF in the ATS/ERS/JRS/ALAT guideline include a usual interstitial pneumonia (UIP) pattern on high-resolution computed tomography (HRCT), characterised by “honeycombing with or without peripheral traction bronchiectasis or bronchiolectasis” with a predominantly subpleural and basal distribution. The same guideline recommends performing a surgical lung biopsy to confirm or refute a diagnosis of IPF in patients with ‘probable UIP’, ‘indeterminate UIP’ or ‘inconsistent with UIP’ patterns on HRCT.

Interestingly, a recent meta-analysis of 12 studies found very little difference in the proportion of confirmed IPF diagnoses in patients with UIP on HRCT compared to patients with possible UIP on HRCT [18]. This suggests that a diagnosis of IPF can be confirmed without surgical lung biopsy in patients with a possible UIP pattern on HRCT. This may have significant implications for patient care and could reduce the burden of the investigations as part of the work-up for IPF diagnosis. As this is a very recent publication, it is likely that we will have to wait to see if and how this is integrated into international clinical guidelines. However, in a recent ‘White Paper’ by the Fleischner Society, Lynch et al. [19] stated that, in the appropriate clinical context, patients with probable UIP on HRCT can avoid having a surgical lung biopsy. The authors justify this recommendation based on studies showing a high level of agreement between probable UIP on HRCT and confirmed UIP on surgical lung biopsy. Instead, the authors suggested that surgical lung biopsy should only be performed if the HRCT pattern is “indeterminate or inconsistent with UIP” or when there are “clinical features to suggest an alternative diagnosis”.

Prior to making a diagnosis of IPF, it is important to carefully consider alternative diagnoses due to the many clinical and radiological similarities between IPF and other interstitial lung diseases [2,20]. Indeed, due to radiological similarities on HRCT, many patients with presumed IPF based on initial clinical evaluation may, in fact, have chronic hypersensitivity pneumonitis (CHP) [21]. Patients may forget having been exposed to occupational and environmental antigens in the past, important factors which may favour a diagnosis of CHP [2]. In a prospective cohort study of 60 patients diagnosed with IPF, based on international guidelines, Morell et al. [22] found that almost half of the patients were subsequently diagnosed with CHP following additional tests and identification of antigen exposures known to cause CHP during the 6-year follow-up period.

## 3. Pathogenesis of IPF

The pathogenesis of IPF is incompletely understood. However, accumulating evidence, obtained over the past three decades, suggests that several risk factors are associated with the development of IPF, including advanced age, male gender, genetic factors, smoking and other environmental exposures [23,24,25,26]. A great deal of attention has been directed towards elucidating the role of genetic factors in the pathogenesis of IPF. Data obtained from mechanistic studies suggests that IPF may arise due to a sequence of adverse pathobiological processes resulting from predisposing factors including genetic mutations, gene–environment interactions, aging and telomere shortening [27]. These predisposing factors are ultimately thought to lead to epithelial dysfunction, promoting profibrotic processes including pathologic fibroblast differentiation and extracellular matrix deposition and remodelling. On a cellular level, IPF is thought to arise due to recurrent alveolar epithelial injury in addition to impaired epithelial repair, leading to increased myofibroblast activity and resultant interstitial fibrogenesis [28,29]. The cumulative clinical manifestation of this cellular process is stiff, fibrotic lungs with decreased lung volumes, decreased gas transfer and a restrictive deficit on spirometry [30].

## 4. Role of Occupational and Environmental Exposures

There is an argument that many patients labelled as having IPF may instead have a form of interstitial lung disease, which has, in fact, been triggered by an occupational or environmental exposure [31]. This would, of course, imply that the patients’ clinical condition is not idiopathic. As discussed previously, many patients are misclassified as having IPF, which may be due to insufficient tests or inadequate occupational and environmental history taking, as highlighted by the study by Morell et al. [22]. A possible alternative explanation may be that clinicians do identify environmental exposures; however, these exposures are not perceived to be of a sufficient intensity to suggest a causal relationship with the patients’ interstitial lung disease.

Examples of occupational and environmental exposures which may contribute to the pathogenesis of IPF include organic dust (livestock/agriculture/farming), metal and mineral dust, wood dust, asbestos and ambient particulate matter [31,32,33,34,35,36,37]. The evidence suggesting a role of occupational and environmental exposures in the pathogenesis of IPF is largely drawn from case-control studies over the past three decades.

A recent case-control study, conducted in southern Italy, found that two groups of occupations conferred a particularly high risk of developing UIP and that this risk increased with increased lengths of occupational exposure: farmers, veterinarians and gardeners (adjusted odds ratio, 2.73; 95% confidence interval, 1.47–5.10) and metallurgical and steel industry workers (adjusted odds ratio, 4.80; 95% confidence interval, 1.50–15.33) [38]. Furthermore, the authors found that people with a self-reported history of exposure to metal dust and fumes and organic dust were at a higher risk for developing UIP. The finding of a dose–response relationship, i.e., a longer time working in a high-risk job was associated with increased risk of IPF in this study, strengthens the hypothesis that occupational exposures play a role in the pathogenesis of IPF. A methodological strength of this study was that the occupational exposure history was not only self-reported by patients. The authors also determined exposure history using a job exposure matrix (JEM), which is less prone to recall bias.

Data obtained from a multi-centre, case-control study in the US identified several occupations which were associated with IPF, including farming, livestock, hairdressing, raising birds, stone cutting/polishing and occupations which gave exposure to metal dust and vegetable dust/animal dust [39]. In a multivariate logistic regression model, adjusted for age and smoking history, the occupations which showed the strongest association with IPF were raising birds (odds ratio, 4.7; 95% confidence interval, 1.6–14.1) and exposure to vegetable dust/animal dust (odds ratio, 4.7; 95% confidence interval, 2.1–10.4). In a more recent analysis of US mortality data, comprising 84,010 deaths due to IPF, Pinheiro et al. [40] identified three occupational groups, with potential exposure to wood and metal dusts, which had increased risk of IFP mortality: “Wood buildings and mobile homes”, “Metal mining” and “Fabricated structural metal products”.

Occupational dust exposure has also been shown to impact prognosis in patients with IPF. In a national survey of 1311 patients with a diagnosis of IPF in Korea, Lee et al. [41] found that dust-exposed workers showed earlier-onset IPF, a longer duration of symptoms at diagnosis and higher mortality than workers without occupational dust exposure. This highlights not only a possible pathobiological link between occupational dust exposure and IPF, but a possible important association with worse clinical outcome in patients with IPF.

## 5. Organic Dust

In addition to data previously mentioned [38,39], several other studies have demonstrated a potential role of organic dust exposure in the development of IPF. In an Egyptian multi-centre case-control study, Awadalla et al. [42] showed that the risk of IPF was higher in females working in farming, raising birds and occupational exposures to animal feeds, products and dusts and pesticides. Conversely, a higher risk of IPF was observed in males working in chemical and petrochemical industries and carpentry and wood working with occupational exposures to wood dust and wood preservatives. The observed between-sex differences may reflect differences in the overall numbers of each sex working in these industries in Egypt. Focussing on individual organic exposures, the authors found that exposure to cats or birds conferred a higher risk of IPF development in both sexes. Furthermore, in both sexes, the risk of IPF was lower in those working in sales and clerical related activities.

In a recent study of 244 patients with MDT-confirmed diagnoses of IPF in Belgium, De Sadeleer et al. [43] separated patients into two groups based on the presence or absence of past exposure to moulds/birds and then compared patients with a third group of patients with CHP. Interestingly, the authors found that the risk of mortality was significantly lower in IPF patients exposed to moulds/birds than those who were not (adjusted hazard ratio, 0.60; 95% confidence interval, 0.37–0.97). A key strength of this study was the robustness of the IPF and CHP diagnoses, which were based on MDT discussion and the international diagnostic guideline criteria. It is unclear why the mould/bird-exposed IPF patients had lower mortality than the non-exposed IPF patients, particularly given that these exposures are thought to play a role in the pathogenesis of IPF in the first place. The main message from this study is that it is possible to separate IPF patients into groups, with different risks of mortality, based on the presence or absence of environmental exposures. As the authors note, it is unclear whether these patients should be considered as a subgroup of IPF or a ‘major fibrotic CHP subgroup’.

## 6. Metal and Mineral Dust

In a recent, comprehensive literature review, Assad et al. [44] evaluated clinical and mechanistic studies to try and determine the role of metals in the development of pulmonary fibrosis. The authors found that occupational and environmental exposure to numerous metals appeared to show an association with pulmonary fibrosis, including aluminium, arsenic, cadmium, copper, molybdenum, tungsten and colbalt, uranium and vanadium. Early case reports suggested a role of occupational aluminium and silica inhalation in the development of pulmonary fibrosis [45,46,47,48,49,50]. Building upon these findings, using archived occupational history data and pension-fund mortality data, Hubbard et al. [51] investigated the role of occupational metal exposure on mortality due to IPF in a cohort of employees of a major UK engineering company. The authors found that, in metal-exposed workers, there was a significant relationship between the duration of metal exposure and the risk of IPF-related death. The findings from this important study suggested that there is a dose-dependant relationship between metal exposure and risk of death in patients with IPF.

Similar findings supporting the role of metal dust inhalation in the pathogenesis of IPF have been observed in Japanese and Korean cohorts. In a multi-centre case-control study in Japan, Miyake et al. [52] found that patients working in clerical jobs had a significantly lower risk of IPF (adjusted odds ratio, 0.42; 95% confidence interval, 0.18–0.95) whereas patients with previous occupational exposure to metal dust had an increased risk of IPF (adjusted odds ratio, 9.55; 95% confidence interval 1.68–181.12). In an autopsy-based study, Kitamura et al. [53] performed histopathological analysis of pulmonary hilar lymph nodes in 23 cases of IPF/UIP and 23 controls derived from a Japanese cohort. The authors found that the lymph nodes of the cases contained significantly more aluminium and silicon and that these deposits were associated with an increased risk of IPF/UIP. The magnitude of the association between silicon and IPF/UIP was particularly high (adjusted odds ratio, 57.84; 95% confidence interval 1.45–2306.19), but may be partly explained by the small numbers and a wide confidence interval. Finally, two small case-control studies conducted in South Korean populations found that occupational and environmental exposure to metal dust [54] and stone, sand, and silica [55] were associated with the development of IPF.

## 7. Wood Dust

Several studies have demonstrated an association between exposure to wood dust and IPF. In an early, UK-based case-control study, Hubbard et al. [56] evaluated the relationship between metal/wood dust and IPF using postal questionnaires, augmented by telephone interviews, to determine occupational exposure history. The authors identified 218 cases and 569 controls and found that exposure to wood dust was associated with a significantly higher risk of IPF (adjusted odds ratio, 1.71; 95% confidence interval, 1.01–2.92). A similar magnitude of association was seen between exposure to metal dust and IPF in this study (adjusted odds ratio, 1.68; 95% confidence interval, 1.07–2.65). Confirming previous findings, the authors of this important study observed a dose–response relationship, with each additional year of occupational exposure conferring a higher risk of IPF. Furthermore, the estimated population attributable risk (PAR) for wood dust and metal dust exposure were 7.1% and 12.5%, respectively. This suggests that, in this study, around 20% of cases of IPF were explained by exposure to wood/metal dust. The implication of this finding is that despite being labelled as a disease of unknown aetiology, IPF may potentially be a preventable disease in some cases and that interventions to reduce high-risk occupational exposures may be a worthwhile public health priority.

In a national, multi-centre case-control study based in Sweden, Gustafson et al. [57] analysed data from a prospective registry of 140 severe IPF patients on long-term oxygen therapy and compared them to 757 age-matched controls selected at random from the general population. Using a postal questionnaire to determine occupational exposure history, the authors found that men with a history of exposure to two specific wood dusts had a higher risk of IPF: birch dust (odds ratio, 2.7; 95% confidence interval, 1.30–5.65) and hardwood dust (odds ratio, 2.7; 95% confidence interval, 1.14–6.52). This relationship was not seen in women, which may be due to the relatively small numbers of women working in occupations with exposure to these dusts. Notwithstanding, the results of this study suggest a possible role of exposure to specific wood dusts in the pathogenesis of IPF.

Additional data supporting the idea that wood dust exposure may play a role in the pathogenesis of IPF has been presented in the form of a case-series from Italy [58] and, as discussed previously, the work from Egypt by Awadalla et al. [42], which found that men with a history of carpentry and wood-working with occupational exposure to wood dust and wood preservative had a significantly higher risk of IPF. These studies suggest that occupational exposures to wood dusts may contribute to the development of IPF. However, further work, in the form of prospective, population-based studies is required to confirm these findings.

## 8. Asbestos

The role of occupational asbestos exposure in the development of asbestos-related lung diseases, such as pleural plaques, asbestosis and mesothelioma, is well established [59]. However, the role of mild to moderate asbestos exposure in the development of IPF is less clear [60].

Barber et al. [61] attempted to explain the relationship between historical UK asbestos importation and mortality in patients with IPF. Using mortality data for England and Wales, obtained from the Office for National Statistics, the authors created linear regression models to quantify the relationship between asbestos importation and IPF mortality. A key finding in this study was that historic asbestos imports shared a significant, positive linear relationship with IPF mortality. The authors tentatively suggest that the observed strength of association between IPF mortality and historic asbestos imports may suggest that a proportion of the IPF related mortality may be due to unrecognised asbestos exposure. It is important to note the limitations of the analysis of asbestos exposure used in this study; the authors calculated total UK asbestos imports per year rather than asbestos consumption per capita and did not determine the cumulative effect of exposure to existing asbestos products. Despite its limitations, this study suggests a possible relationship between exposure to asbestos and IPF mortality in the UK.

The IPF Job Exposure Study (IPF-JES) is an ongoing, UK, national, prospective case-control study, which will provide important new information about the association (or lack of association) between occupational asbestos exposure and the development of IPF (https://clinicaltrials.gov/ct2/show/NCT03211507). Studies like IPF-JES, which help to determine a relationship between asbestos exposure and risk of IPF, are important. This is particularly true given that the rate of asbestos consumption remains high in many developing countries [62].

## 9. Ambient Particulate Matter

More recently, there has been a focus on the role of ambient particulate matter, including background air pollution, in the pathogenesis of IPF. In the first study of its kind, Conti et al. [63] investigated the relationship between chronic exposure to ambient air pollutants (ozone (O3), nitrogen dioxide (NO2) and particulate matter with an aerodynamic diameter <10 μm (PM10)) and incidence of IPF in an Italian population. The authors used administrative databases to calculate regional IPF incidence in Northern Italy and modelled the association between regional IPF incidence and average ambient air pollutant concentrations. The authors observed a statistically significant relationship between overall NO2 exposure and IPF incidence. However, this study has several limitations and it is important to note that the analyses did not adjust for important IPF risk factors, including smoking [64].

In an earlier study, Johannsen et al. [65], investigated the relationship between air pollution and acute exacerbations of IPF. The authors found that increased exposure to O3 and NO2 over the preceding 6 weeks, was associated with increased risk of acute exacerbation of IPF. Three further studies, published in the last two years, have also assessed the relationship between ambient particulate matter exposure and clinical outcomes in patients with IPF [66,67,68].

Winterbottom et al. [66] studied 135 patients with IPF and found that exposure to ambient particulate matter with an aerodynamic diameter <10 μm (PM10) was associated with an increased rate of decline in lung function, as measured by the forced vital capacity (FVC). Conversely, the authors did not demonstrate a relationship between exposure to particulate matter with an aerodynamic diameter <2.5 μm (PM2.5) and decline in lung function. Furthermore, this study did not identify a significant relationship between exposure to PM10 or PM2.5 and mortality in patients with IPF. In a smaller, prospective study of 25 patients with IPF, Johannsen et al. [68] failed to demonstrate a relationship between the ambient air pollution (O3, NO2, PM10 and PM2.5) and the rate of decline of lung function as measured by FVC over the 40-week study period. However, the negative findings of this study may, in part, be due to the small sample size and must be interpreted with caution.

Sesé et al. [67] investigated the impact of ambient air pollution (O3, NO2, PM10 and PM2.5) on the clinical course of IPF, using a prospective, longitudinal French cohort of 192 IPF patients. A multivariate Cox proportional hazards model found that exposure to O3 was associated with an increased risk of acute exacerbations of IPF, whilst exposure to PM10 and PM2.5 was associated with increased mortality. The study by Sesé et al. [67] is the largest to date and has several methodological strengths, as noted in a commentary by Johannson et al. [69]. Taken together, these studies suggest that environmental exposure to ambient particulate matter may play a role in both the pathogenesis and the progression of IPF in patients with established disease, and that this is an important area for future research [70].

## 10. The Challenge of Identifying Occupational and Environmental Exposures

Clearly, there is a need to complete a detailed occupational and environmental exposure history in the work-up for the diagnosis of IPF. This can be a difficult endeavour for several reasons. Firstly, patients may simply not recall certain occupational or environmental exposures, or may not have known they were being exposed, particularly in older cohorts before the stricter health and safety requirements came into force. Secondly, it can be difficult to estimate the intensity of an exposure and how this may have varied over time. Calculations, which provide an estimate of lifetime exposure and the cumulative burden of exposure, may provide valuable data but may be practically challenging to perform due to heterogenous levels of exposure in the ‘real world’ [71]. It may also be difficult to accurately determine the magnitude of the relationship between specific exposures and the development of IPF due to multiple exposures occurring simultaneously [72]. Finally, it is important to note that the case-control design of many of the epidemiological studies investigating the role of past occupational and environmental exposures is subject to recall bias. Recall bias may influence the results of these studies, particularly in patients with IPF who may be more inclined to extensively search for possible reasons to explain their condition. The development of job-exposure matrices, specific to occupational exposures such as asbestos, can facilitate a more robust and reproducible assessment of lifetime occupational exposure [73]. Prospective, longitudinal, population-based cohort studies, which accurately assess occupational and environmental exposures at baseline and identify the association between these exposures and IPF, are needed. Prospective cohorts would build upon the existing retrospective case-control data and would provide more robust data regarding the relationship between specific exposures and IPF.

## 11. Concluding Remarks

As our understanding of the pathogenesis and clinical course of IPF develops, we are likely to see further changes in the case definition and possible subclassifications [74]. Furthermore, as particular phenotypes with clearer pathobiological causes are identified, we may move away from labelling patients’ conditions as ‘idiopathic’ and may even see diagnoses reclassified using alternative nomenclature [75]. This remains a controversial topic in the IPF community and it remains to be seen whether the optimal course of action will be retaining the terminology of IPF and subclassifying patients or merging IPF diagnoses with other fibrotic lung diseases [74]. An accumulating body of evidence suggests that occupational and environmental exposures play a role in the development of IPF and this is an area which warrants further investigation. The correct identification of the underlying causes of fibrotic lung disease has important implications for disease management and prognosis.
